# Distribution and Control of Bacterial Community Composition in Marian Cove Surface Waters, King George Island, Antarctica during the Summer of 2018

**DOI:** 10.3390/microorganisms8081115

**Published:** 2020-07-24

**Authors:** Soyeon Kim, Ju-Hyoung Kim, Jae-Hyun Lim, Jin-Hyun Jeong, Jang-Mu Heo, Il-Nam Kim

**Affiliations:** 1 Department of Marine Science, Incheon National University, Incheon 22012, Korea; soyeonkim@inu.ac.kr (S.K.); jangmuheo@inu.ac.kr (J.-M.H.); 2 Faculty of Marine Applied Biosciences, Kunsan National University, Gunsan 54150, Korea; 3 Fisheries Resources and Environmental Research Division, East Sea Fisheries Research Institute, National Institute of Fisheries Science, Gangneung 25435, Korea; lim900@korea.kr; 4 Korea National Ocean Science Museum, Uljin 36315, Korea; pujjh@hotmail.com

**Keywords:** Antarctic, Marian Cove, bacterial community composition, environmental changes

## Abstract

Marian Cove is experiencing some of the most rapid environmental changes in the Antarctic region; however, little is known about the response of bacterial communities to these changes. The main purpose of this study was to investigate the spatial variation of physical–biogeochemical–bacterial community features in the Marian Cove surface waters and the environmental parameters governing the spatial variation in the bacterial community composition during the summer of 2018. The Marian Cove surface waters are largely composed of two different characteristics of water masses: relatively low-temperature, -salinity, and -nutrient surface glacier water (named SGW) and relatively high-temperature, -salinity, and -nutrient surface Maxwell Bay water (named SMBW). The SGW bacterial communities were dominated by unclassified Cryomorphaceae, Sedimenticola, and Salibacter genera, while the SMBW bacterial communities were dominated by Sulfitobacter, Arcobacter, and Odoribacter genera. Spatial variations in bacterial community composition were mainly attributed to physical and biogeochemical characteristics, suggesting that the bacterial community composition of the Marian Cove surface waters is mainly determined by environmental characteristics. These findings provide a foundation to improve the understanding of bacterial community variations in response to a rapidly changing Marian Cove in the Antarctic.

## 1. Introduction

Antarctic regions have played an important role in regulating the earth’s climate system, not only on a glacial–interglacial timescale but also during the Anthropocene era [1,2]. In spite of harsh environmental conditions, such as low temperatures and extreme seasonal variations, the Antarctic aquatic ecosystem has shown active and diverse microbial communities [3], driving biogeochemical cycles and supporting higher trophic levels. Recently, Antarctic regions have been undergoing rapid warming at alarming rates [4], which is a serious threat to ice-based polar ecosystems. In particular, the water temperature of the western Antarctic Peninsula has risen by about 2 °C since 1950, which has had a significant effect on the reduction of the total icy area of the peninsula [5,6].

**Figure 1:**
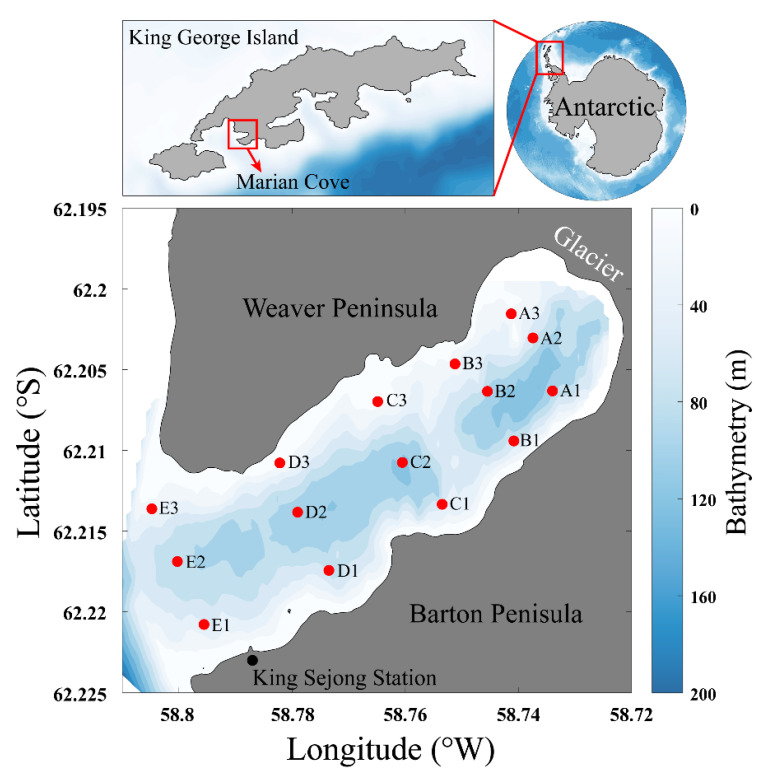
Map showing the sampling locations in Marian Cove, King George Island, Antarctic. The color bar indicates water depth in meters.

Marian Cove, which is located between Weaver and Barton Peninsulas (Figure 1), is one of the regions of the western Antarctic Peninsula where rapid environmental changes have occurred. In particular, Marian Cove has experienced a rapidly increased inflow of freshwater as a result of substantial glacier retreat (decline of 1.7 km since 1956 in summertime) [7,8,9,10,11], leading to the prediction that strong stratification will form and, in turn, result in a shift to a nutrient-limited ecosystem [12]. Previous studies pointed out that increasing freshwater inflow can alter environmental characteristics and change the community composition of living organisms (e.g., algae and benthic species) in Marian Cove [13,14,15,16,17,18]. In addition, it was reported that the bacterial community was significantly different within Maxwell Bay [19,20], possibly reflecting that bacterial community composition is substantially influenced by surrounding physical and biogeochemical properties [13,16,17].

Given that studies about the variations of microbial communities in various environments have profoundly increased our understanding of the responses of bacterial communities to environmental changes [17], Marian Cove can be considered an appropriate place to investigate the impact of climate change on Antarctic aquatic ecosystems via investigations of variation in bacterial communities. However, little is known about even the spatial distribution of the bacterial community and its control factors in Marian Cove to date. To predict the response of Marian Cove aqueous ecosystems to ongoing environmental changes, an intensive investigation of the spatial variation in bacterial community composition and its controlling physical and biogeochemical factors is important.

Therefore, the main purpose of this study was, for the first time, to (1) present the spatial distributions of physical–biogeochemical–bacterial community features in Marian Cove surface waters during summer, (2) investigate the environmental parameters governing the spatial variation in the bacterial community composition, and (3) provide significant insight into the alteration of the Marian Cove bacterial community in response to rapid Antarctic environmental changes.

## 2. Materials and Methods

### 2.1. Sample Collection

Marian Cove is a small cove, 4.5 km in length, 1.5 km in width, and up to 120 m deep (Figure 1 and Table 1) [7]. Marian Cove is characterized by the continuous inflows of seawater and freshwater [21]. Seawaters enter Marian Cove via Maxwell Bay along the western Antarctic Peninsula in the Bellingshausen Sea [22]. In January 2018, surface seawaters (at ~1 m depth) were collected from 15 different stations located between the glacier present inside Marian Cove and Maxwell Bay using 5 L Niskin bottles (Table 1). For bacterial community analysis, 2 L of seawater in each station was filtered through a 0.2-μm membrane (Whatman 47 mm polycarbonate membrane) to capture the microbial cells. Filtered samples were then immediately frozen and stored at −80 °C until DNA extraction.

### 2.2. Measurement of Physical and Biogeochemical Parameters

Physical and biogeochemical parameters of the water samples were measured at each station. Water temperature (T), salinity (S), and dissolved oxygen were measured using a conductivity–temperature–depth instrument (CTD; RBR Ltd., Ottawa, ON, Canada). For nutrient analysis, the sample at each station was filtered through a 0.2-µm syringe filter (Sartorius, Cat. No 16532), placed in a 50-mL conical tube, and stored at −20 °C until measurement. After thawing the frozen seawater, phosphate (PO_4_), nitrate and nitrite (NO_3_ + NO_2_), silicate (SiO_2_), and ammonium (NH_4_) were measured using an autoanalyzer (Quaatro, Seal Analytical, Germany). Dissolved inorganic nitrogen (DIN) is represented as the sum of NH_4_ + NO_3_ + NO_2_.

### 2.3. DNA Extraction, 16S rRNA Gene Amplification, and Sequencing

To assess the bacterial community composition, DNA from the filter paper was extracted using the PowerSoil^®^ DNA Isolation Kit (Cat. No 12888, MOBIO), according to the manufacturer’s protocol. Quantitative and qualitative analyses of DNA were carried out using PicoGreen and Nanodrop, respectively. Polymerase chain reaction (PCR) was carried out to amplify the V3–V4 regions from the extracted environmental DNA, a variable sequence of the 16S rRNA gene, using primers 341F and 805R [23]. The PCR products were standardized using PicoGreen, and the library size was verified using a TapeStation DNA screen tape D1000 (Agilent). Sequencing was performed using the MiSeq™ platform (Illumina, San Diego, CA, USA). The paired-end sequence generated as a result of the sequencing was merged using FLASH (v. 1.2.11) to obtain a single long sequence [24]. The sequence was obtained by removing low-quality sequences, ambiguous sequences, and chimera sequences, which are regarded as sequencing errors; denoising by using the operational taxonomic unit (OTU) analysis program CD-HIT-OTU, and then clustering sequences with 97% or more sequence similarity at the species level of OTU [25]. QIIME (v. 1.8.0) was used to analyze microbial populations (Alpha and Beta Diversity, Multiple Alignment, Phylogeny), and each OTU was compared to the National Center for Biotechnology Information (NCBI) DB (16S Microbial DB) [26]. Raw sequence data were deposited in the NCBI with Sequence Read Archive (SRA) accession number PRJNA533713. Finally, a total of 650,487 reads were obtained from 15 samples and were clustered into 1467 OTUs (Table 1).

### 2.4. Statistical Analysis

To analyze the relationship between stations and environmental parameters, principal component analysis (PCA) was performed using the R stats package (v. 3.5.2, http://www.r-project.org/) [27]. Permutational multivariate analysis of variance (PERMANOVA) was used to investigate grouping from the relationship between bacterial community composition and environmental parameters using the PERMANOVA+ in Primer6 (v. 6.1.16) program [28] and then visualized on principal coordinates analysis (PCoA) ordination using the Primer6 (ver. 6.1.16) program [29]. Heatmaps were visualized using the R packages (i.e., Heatplus v. 1.3, vegan v. 2.5-6, gplots v. 3.0.1.1, and RColorBrewer v. 1.1-2), and, for clustering analysis, Bray-Curtis dissimilarity was used to determine similarity among bacterial communities using the R package vegan (v. 2.5-6) [30,31]. The indicator species analysis was generated using R package indicSpecies (ver. 1.7.9). Spearman’s rank–order correlation coefficients were calculated for non-normally distributed data (i.e., between the relative abundance of bacterial compositions and physical and biogeochemical variables) using the R package vegan (ver. 2.5-6) [32,33].

## 3. Results and Discussion

### 3.1. Physicochemical Characteristics and Bacterial Community Composition

**Figure 2:**
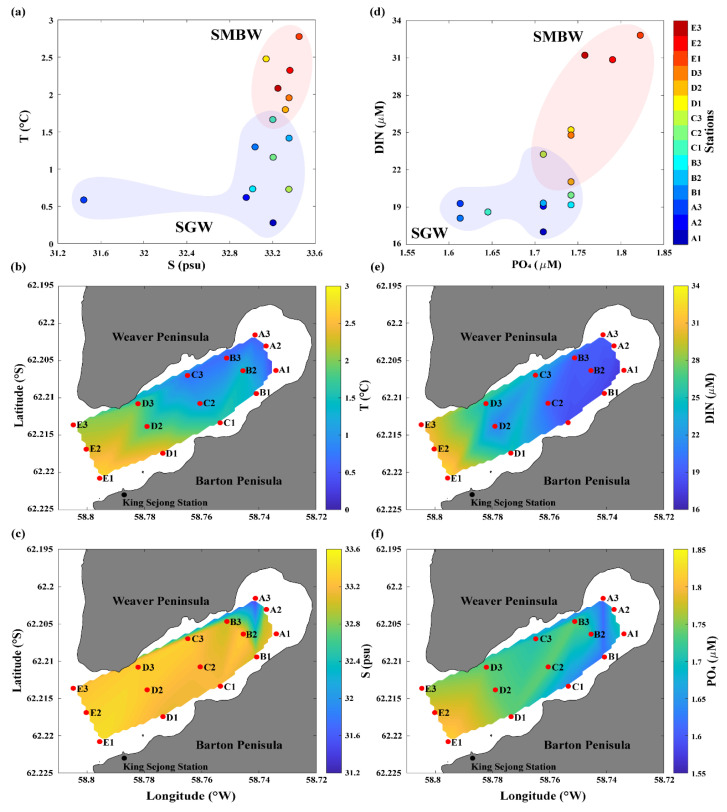
Physical and biogeochemical properties of the Marian Cove surface waters during the summer of 2018: (**a**) temperature (T)–salinity (S) diagram, spatial distribution of (**b**) T and (**c**) S, (**d**) dissolved inorganic nitrogen (DIN)–phosphate (PO_4_) plot, and the surface distribution of (**e**) DIN and (**f**) PO_4_.

We used T and S data collected in Marian Cove during the summer of 2018 to investigate the physical characteristics of surface waters on a T–S diagram (Table 1 and Figure 2a). Based on the data distribution pattern, two different water masses were found to be involved in the mixing process in the study area: relatively low T and S water (named surface glacier water, SGW) and relatively high T and S water (named surface Maxwell Bay water, SMBW). The mean T and S values of surface waters were relatively low in the SGW region (~1.0 °C and 32.8, respectively) compared with those in the SMBW region (~2.2 °C and 33.3, respectively) (Figure 2b,c). SGW covered the regions of A1–C3, and SMBW covered the regions of D1–E3 (Figure 1 and Figure 2b,c). The observation of lowest S in A3 could be because the glacier collapsed at the time of sampling (Figure 2a,c), and the debris flowed directly into the surface Marian Cove waters. The physical properties of Marian Cove surface waters showed a gradual physical gradient between the cold and fresh SGW and warm and saline SMBW (Figure 2a–c).Surface waters also exhibited a nutrient gradient similar to the physical parameters in Marian Cove (Figure 2d). Differences in DIN and PO_4_ concentrations were also apparent between the SGW and SMBW regions (Figure 2e,f). The mean DIN and PO_4_ concentration were relatively low in the SGW region (~19.3 and 1.69 μM, respectively) compared with those in the SMBW region (~27.7 and 1.77 μM, respectively) (Figure 2e,f). In summary, the SGW region is characterized as having relatively low T, S, and nutrients, while the SMBW region has relatively high T, S, and nutrients. Therefore, the Marian Cove surface water samples showed a physical and biogeochemical gradient between the SGW and SMBW regions (Figure 2).

**Figure 3:**
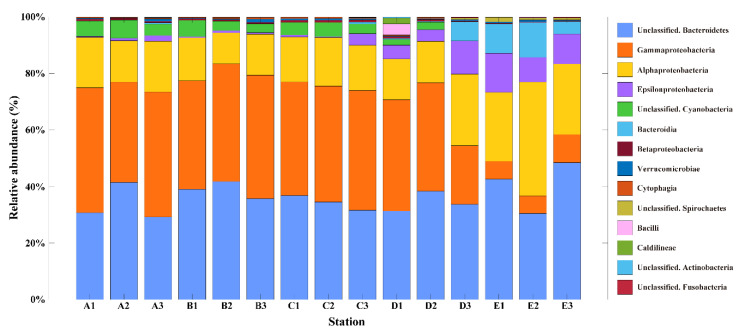
The relative abundance (%) of the surface Marian Cove bacterial communities at the class level during the summer of 2018. Note that taxa occurred at >1 % in a total sum of relative abundance of all stations were represented.

To characterize the difference in bacterial community compositions with space, the relative abundance (%) of bacterial communities at the class level was analyzed (Figure 3). In the SGW regions, Gammaproteobacteria (mean: 41%), unclassified Bacteroidetes (mean: 35%), Alphaproteobacteria (mean: 15%), and unclassified Cyanobacteria (mean: 5%) were the dominant classes, accounting for >90% of the relative abundance (Figure 3). Meanwhile, five classes of unclassified Bacteroidetes (mean: 37%), Alphaproteobacteria (mean: 24%), Gammaproteobacteria (mean: 20%), Epsilonproteobacteria (mean: 9%), and Bacteroidia (mean: 6%) were the dominant classes in the SMBW region, accounting for >90% of the relative abundance (Figure 3). Alphaproteobacteria and Gammaproteobacteria are known to be predominant in the ocean, and Betaproteobacteria are predominant in freshwaters, such as rivers and lakes [34,35]. The relative abundance results of this study confirmed that the bacterial taxonomy was different in the SGW and SMBW stations.

**Figure 4:**
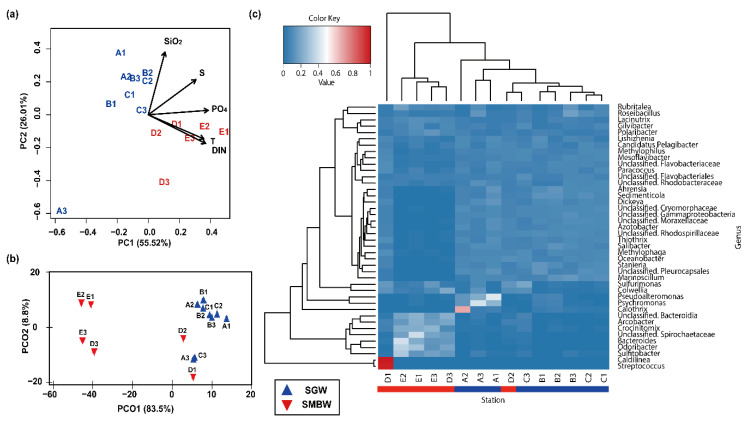
(**a**) Principal component analysis (PCA) ordination biplot of sampling stations with environmental parameters (physical: T and S; biogeochemical: DIN, PO_4_, and SiO_2_). The two components (PC1 and PC2) explained 73.46% of the total variation in environmental data. (**b**) Principal coordinates analysis (PCoA) ordination plot of Bray–Curtis dissimilarity calculated from the relationship between bacterial community composition at the genus level and environmental parameters using PERMANOVA analysis (F statistic = 15.6 with *p* < 0.001). (**c**) Heatmap shows the relative abundance at the genus level (>1 % in a total sum of relative abundance of all stations), and the dendrogram shows the hierarchical clustering of each bacterial taxa and station.

Based on PCA (Figure 4a), the stations were well divided into two distinct groups of SGW and SMBW; SGW stations were closely linked to SiO_2_, while SMBW stations were associated with T, S, DIN, and PO_4_. In line with PCA, PCoA also showed that the bacterial community composition along the stations was likely to be distinctly separated into SGW and SMBW groups (Figure 4b). In addition, the investigation of the difference in dominant phylogenetic bacterial taxa at the genus level for each station revealed that they were well clustered into SGW and SMBW groups (Figure 4c), except for the D2 station. Overall, these results suggest that the environmental characteristics of the surface of the Marian Cove waters played an important role in determining the surface Marian Cove bacterial community composition during the summer of 2018 [19,20,36].

Uniquely, a high relative abundance of Streptococcus (3.6%), which commonly inhabits the guts of Antarctic penguins and birds [37,38], was found at the D1 station (Figure 4c). This station is likely to be influenced by the influx of excrements from a variety of terrestrial organisms and shows the greatest richness and diversity of bacterial taxa (Table 1).

**Figure 5:**
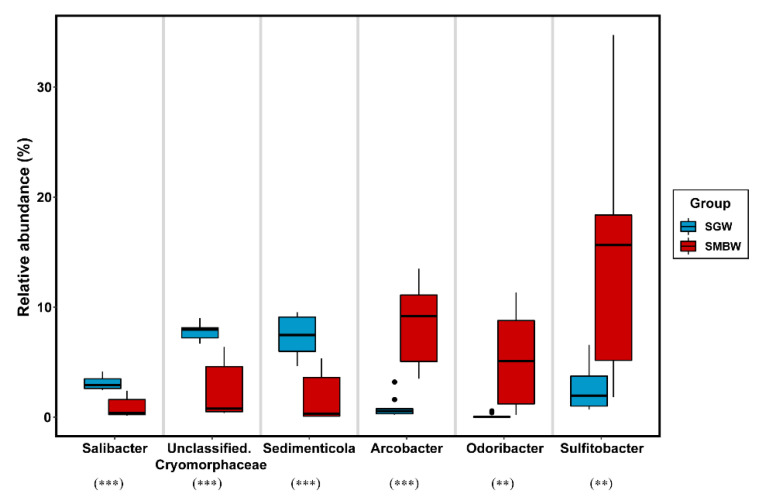
Boxplots to represent indicator species analysis at the genus level in the SGW and SMBW groups. Filled dots represent outliers. The symbols of ** and *** indicate a *p* value of less than 0.01 and 0.001, respectively.

Polaribacter (of the class Bacteroidia) was one of the dominant bacterial groups in the surface of the Marian Cove waters (Figure 4c). According to indicator species analysis (Figure 5), Salibacter, unclassified Cryomorphaceae, and Sedimenticola were relatively dominant in the SGW region, whereas Arcobacter, Odoribacter, and Sulfitobacter were relatively dominant in the SMBW region. The relative abundance of Sulfitobacter (family Rhodobacteraceae), which are known to play a role in the degradation of dimethylsulfoniopropionate (DMSP) to dimethyl sulfide (DMS) in the sulfur pathway [39,40], varied from 1.9% to 34.7% in the study area, but was higher in the SMBW region (14.8%) than in the SGW region (2.5%), indicating that decomposition of organic matters is likely to be more active in the SMBW region than in the SGW region.

**Figure 6:**
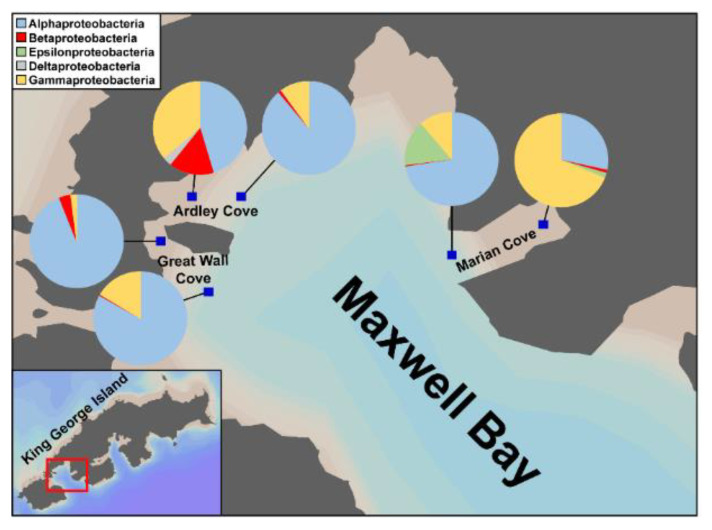
Comparison of bacterial community composition in Antarctic Ardley, Great Wall, and Marian Coves. Pie charts show the relative abundance of bacterial communities at the class level (associated with the phylum level of Proteobacteria) at each location (blue squares). Note that the Ardley and Great Wall Coves data are reproduced from the results of previous study [20] and the Marian Cove data are from the results of this study.

It is known that alteration of bacterial community composition is attributed to environmental changes [23,41,42]. Ardley and Great Wall Coves, having similar physical properties to the neighboring Marian Cove, are also experiencing increased freshwater inflows due to rapid glacier retreat [20]. The primary dominant bacteria were Alphaproteobacteria and Gammaproteobacteria, but Betaproteobacteria were commonly found in the inner regions of Ardley, Great Wall, and Marian Coves (Figure 6), which are influenced by freshwater inputs (Figure 6). This result supports the claim that the inflow of freshwater from melting glaciers has a significant impact on the surface bacterial community composition. Thus, if glacier retreat is accelerated due to global warming and climate change, the alteration of the bacterial community composition in the regions where glacier retreat takes place in polar marine environments will be obvious.

### 3.2. Environmental Factors Determining Bacterial Community Compositions

**Figure 7:**
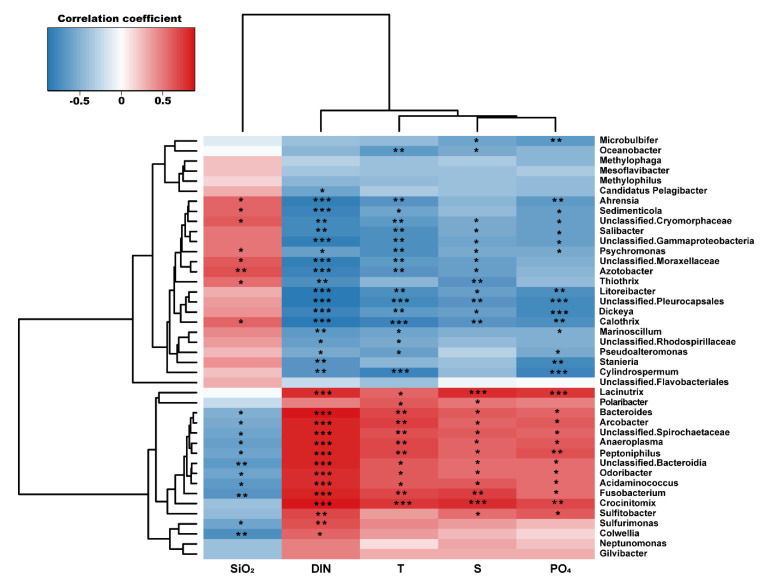
Heatmap from Spearman correlations between environmental parameters (physical: T and S; biogeochemical: DIN, PO_4_, and SiO_2_) and the bacterial community at the genus level (>1% in a total sum of relative abundance of all stations). The symbols *, **, and *** indicate a *p*-value less than 0.05, 0.01, and 0.001, respectively.

In order to investigate the influence of environmental factors on surface bacterial community composition at the genus level, we conducted a correlation analysis between taxa occurred at >1% in a total sum of relative abundance of all stations and physical and biogeochemical parameters (physical: T and S; biogeochemical: DIN, PO_4_, and SiO_2_) (Figure 7). Unclassified Cryomorphaceae, Sedimenticola, and Salibacter, which are dominant in the SGW region, showed significant negative correlations with T, S, DIN, and PO_4_, suggesting that the SGW bacterial compositions were associated with relatively cold, fresh, and less eutrophic conditions. Dominant genera in the SMBW region were Sulfitobacter, Arcobacter, and Odoribacter, which exhibited significant positive correlations with physical and biogeochemical parameters, suggesting that the SMBW bacterial compositions were mainly influenced by the variability of physical properties and nutrient conditions [43]. These results support the claim that the composition of bacterial communities in Marian Cove surface waters is largely determined by environmental characteristics.

### 3.3. Implication for the Surface Marian Cove Bacterial Community Change

Based on the results of this study, at present the marine bacterial community composition (e.g., Sulfitobacter, Arcobacter, and Odoribacter at the genus level) was more dominant than the freshwater composition (e.g., Sedimenticola and Salibacter at the genus level) in the Marian Cove surface waters. Rapid environmental changes in the Antarctic will directly and indirectly affect Antarctic bacterial community compositions. Marian Cove is one of the Antarctic regions undergoing rapid environmental changes due to global warming [15]. In particular, rapid glacier retreat has been found in Marian Cove [7,8,9,10,11,44,45,46,47,48,49], leading to an increase in the inflow of freshwaters into the cove. This phenomenon will, in turn, form strong vertical stratification, limiting the supply of nutrients into the euphotic zone and ultimately lowering primary production. As a result, in the future, the surface Marian Cove bacterial community composition may be shifted from marine to freshwater-dominated genera as a result of freshening environmental change. Such future changes may occur in a number of coves of the Antarctic. Therefore, there is an urgent need to examine how these changes will ultimately affect the Earth’s climate system.

## 4. Summary and Conclusions

We investigated physical–biogeochemical properties and bacterial community composition observed during the summer of 2018 in the Marian Cove surface waters. The SGW region is characterized as having relatively low T, S, and nutrients, while the SMBW region has relatively high T, S, and nutrients. Thus, the Marian Cove surface water samples showed a physical and biogeochemical gradient between the SGW and SMBW regions. In the SGW region, unclassified Cryomorphaceae, Sedimenticola, and Salibacter at the genus level were the dominant groups, and Sulfitobacter, Arcobacter, and Odoribacter genera were dominant in the SMBW region. Overall, the SGW bacterial community composition showed negative correlations with environmental parameters, whereas the SMBW bacterial community composition was positively correlated with environmental parameters. This contrast supports that the bacterial community composition of the Marian Cove surface waters is significantly determined by environmental characteristics. Therefore, attention should be paid to how the Marian Cove bacterial community will be altered in the future in response to rapidly changing Antarctic environments.

## Figures and Tables

**Table 1:** Information of sampling locations, physicochemical properties, number of reads, operational taxonomic unit (OTU), richness (Chao1), and diversity (Shannon).

Station	Latitude (°S)	Longitude (°W)	T (°C)	S (psu)	DIN (μM)	PO_4_ (μM)	SiO_2_ (μM)	Read Counts	OTU	Chao1	Shannon
A1	62.2063	58.7340	0.28	33.20	17.01	1.71	64.44	39,598	235	269.2	4.62
A2	62.2030	58.7374	0.62	32.95	19.07	1.71	63.55	49,976	241	296.1	4.40
A3	62.2016	58.7413	0.58	31.44	19.29	1.61	53.67	35,442	252	290.3	4.75
B1	62.2094	58.7408	1.30	33.04	18.11	1.61	61.48	40,813	228	299.9	4.24
B2	62.2064	58.7455	1.41	33.35	19.32	1.71	63.88	41,502	223	262.0	4.44
B3	62.2047	58.7512	0.73	33.01	19.18	1.74	63.40	33,969	227	280.1	4.53
C1	62.2133	58.7535	1.66	33.20	18.61	1.65	62.45	33,642	243	284.8	4.52
C2	62.2108	58.7605	1.16	33.20	19.96	1.74	63.28	45,573	233	258.2	4.49
C3	62.2070	58.7649	0.73	33.35	23.25	1.71	59.24	44,372	255	298.7	4.78
D1	62.2174	58.7735	2.48	33.14	25.21	1.74	62.33	26,430	300	343.6	5.16
D2	62.2138	58.7790	1.80	33.32	21.04	1.74	57.88	44,444	254	296.0	4.50
D3	62.2108	58.7822	1.95	33.35	24.79	1.74	52.78	53,793	246	299.0	3.76
E1	62.2208	58.7956	2.78	33.45	32.82	1.82	62.75	47,913	248	303.3	3.62
E2	62.2169	58.8003	2.32	33.36	30.86	1.79	62.12	49,610	223	271.4	3.41
E3	62.2136	58.8048	2.08	33.25	31.21	1.76	61.28	63,410	233	296.2	3.31
